# ABC: software for interactive browsing of genomic multiple sequence alignment data

**DOI:** 10.1186/1471-2105-5-192

**Published:** 2004-12-08

**Authors:** Gregory M Cooper, Senthil AG Singaravelu, Arend Sidow

**Affiliations:** 1 Department of Genetics, Stanford University, Stanford, CA 94305-9010, USA; 2 Department of Pathology, Stanford University, Stanford, CA 94305-5324, USA

## Abstract

**Background:**

Alignment and comparison of related genome sequences is a powerful method to identify regions likely to contain functional elements. Such analyses are data intensive, requiring the inclusion of genomic multiple sequence alignments, sequence annotations, and scores describing regional attributes of columns in the alignment. Visualization and browsing of results can be difficult, and there are currently limited software options for performing this task.

**Results:**

The Application for Browsing Constraints (ABC) is interactive Java software for intuitive and efficient exploration of multiple sequence alignments and data typically associated with alignments. It is used to move quickly from a summary view of the entire alignment via arbitrary levels of resolution to individual alignment columns. It allows for the simultaneous display of quantitative data, (e.g., sequence similarity or evolutionary rates) and annotation data (e.g. the locations of genes, repeats, and constrained elements). It can be used to facilitate basic comparative sequence tasks, such as export of data in plain-text formats, visualization of phylogenetic trees, and generation of alignment summary graphics.

**Conclusions:**

The ABC is a lightweight, stand-alone, and flexible graphical user interface for browsing genomic multiple sequence alignments of specific loci, up to hundreds of kilobases or a few megabases in length. It is coded in Java for cross-platform use and the program and source code are freely available under the General Public License. Documentation and a sample data set are also available .

## Background

Functional elements in a genome accumulate inter-specific substitutions more slowly than neutral DNA throughout evolution [1]. Therefore, comparing orthologous genomic sequences from related species is useful for the identification of elements that play important roles in the biology of an organism [2-7]. While the statistical and computational methods for extracting comparative information are variable, the types of data involved are generally quite similar. First, a multiple sequence alignment is necessary. Second, a vector of quantitative scores is produced that describes the similarity of the nucleotides observed in small windows, or individual columns, of the alignment; percent identity is the metric used by the popular program VISTA [8], while a variety of other scoring methods also exist [9-12]. Third, annotations are generated that highlight regions of the alignment that are under constraint or meet some other quantitative threshold. Fourth, annotations of features like transcripts, promoters, coding exons, and repeats provide functional context. Finally, the phylogenetic tree that relates the aligned sequences is important for both performing comparative analyses and for interpreting their results.

Simultaneous visualization of complex data such as these is of utmost importance both for experimentalists and for computational biologists. Several options currently exist for such visualization, but there are a variety of characteristics that distinguish the ABC from them. VISTA, for example, generates a static image that is not interactive [8]. Other popular browsers such as phylo-VISTA [13] and PipMaker [14,15] require the use of a particular alignment program and scoring scheme. Also, the ABC is not suited for genome-wide visualization. Other tools exist for this and are quite useful for the browsing of very large genomic intervals and major evolutionary events such as genomic rearrangements [16-19]. However, these programs are generally part of larger, more complex interfaces that are not necessarily ideal for targeted analysis of an individual alignment or genomic locus. Finally, we note that the ABC allows many annotation types and colors, is not web-based and can be used on a local machine as intensively as necessary, and the source code is open and freely available allowing users to modify and add features if desired.

## Implementation

**Figure 1:**
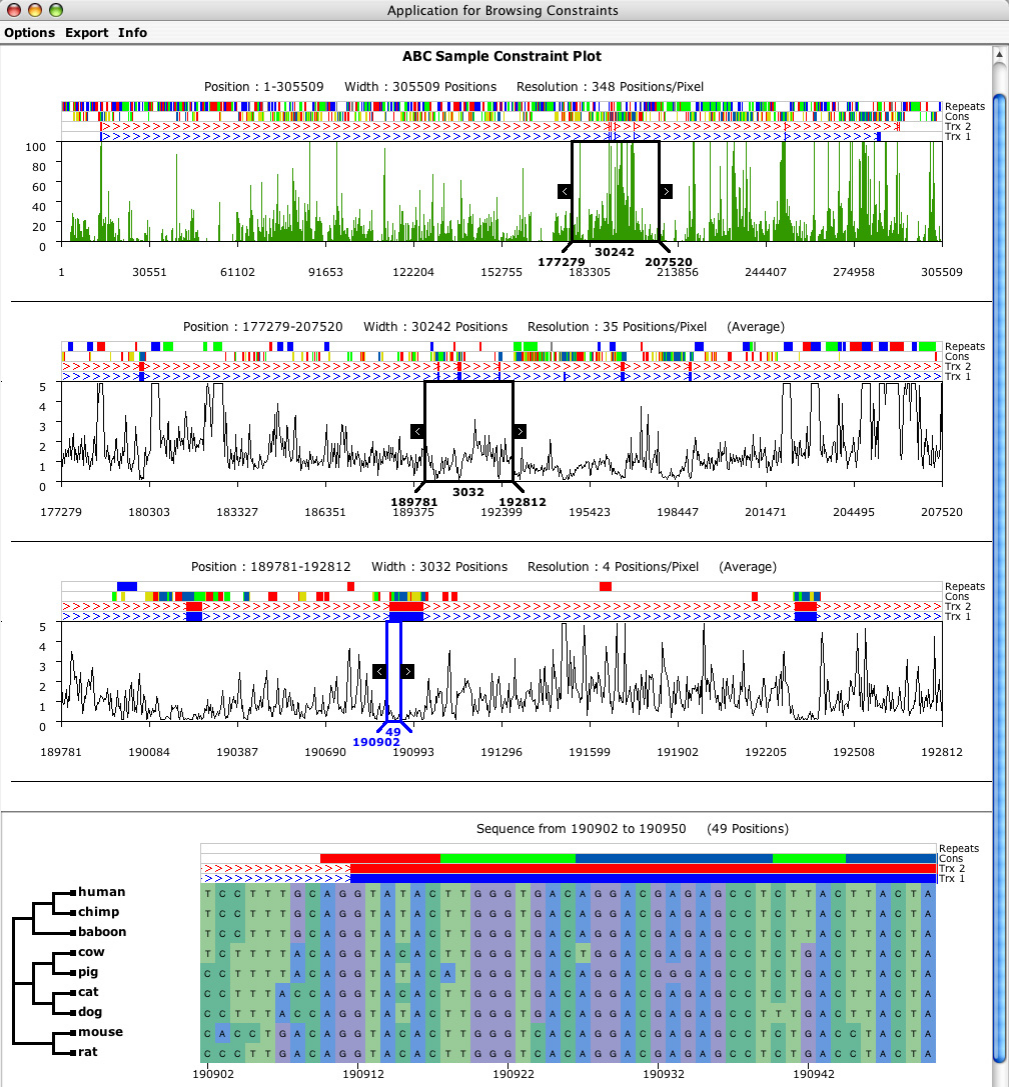
A screenshot of the ABC. The upper-most panel consists of a histogram describing the regional density of columns with low scores, corresponding to regions of high sequence similarity, across the length of the entire alignment. Note the association of peaks in sequence similarity near the exons annotated in the lowest tracks ('Trx 1' and 'Trx 2'). The middle panels consist of 'wiggly plots' of the alignment scores; note the association in these panels between valleys in the plot and the location of exons. The black rectangle can be moved and resized to highlight particular regions. The panels represent increasingly zoomed in views of the regions highlighted within the rectangle, moving from 348 alignment columns per pixel at the top to 4 positions per pixel in the third panel. The bottom panel shows a view of individual alignment columns corresponding to a small region of the alignment. If supplied, a topology describing the relationships of the aligned sequences is also displayed here. Above all panels, four annotation tracks are displayed, the bottom two being reserved for transcripts with exons and introns. In this image, the third and fourth tracks reveal conserved regions and repeats, respectively.

The ABC requires Java 1.4 or later and has been successfully tested on Windows, Linux, and OS X. There is no specific upper limit in the size of potential data sets, but system memory usage can be high on large alignments. The ABC can efficiently handle a 2 Mb alignment of 29 sequences on a machine with a 1 GHz processor and 1 Gb of RAM. Details about file formats and instructions for use are available in the documentation that is available along with the source code . The file formats used by the ABC are quite similar, with only minor modifications, to other standard formats, such as fasta-formatted sequence files and standard parenthesis notation for phylogenetic tree descriptions. A sample data set is available, and is the source of the screenshot depicted in Figure 1. The sequence data are derived from a previously published analysis [20]; it includes ~300 kb of sequence from 9 mammals, centered around the *ST7 *gene in the human genome, near the *CFTR *gene. Repeats were identified in the human sequence using RepeatMasker [21] and genes identified using RefSeq annotations from the UCSC genome browser [17]. The alignment was generated using MLAGAN [22], and has been compressed so that the human sequence is ungapped; annotation of human sequence features is thus identical to the alignment annotations shown in Figure 1. A description of the method used to score the alignment columns and identify constrained elements, along with Perl scripts that facilitate this method, including export of results in ABC-ready formats, will be described elsewhere (in preparation). Please note that the ABC will not translate coordinates from alignment to sequence coordinates (or vice versa); the annotations that the user supplies must be appropriate to the alignment being analyzed.

## Results and discussion

By default, the ABC displays graphical summaries of the quantitative information associated with the alignment. Scores are summarized regionally in consecutive non-overlapping windows. The size of these windows depends on the resolution, defined as the number of alignment columns summarized per pixel. The ABC has three distinct display modes, chosen automatically depending on the density of the information. At very low resolution, a histogram is displayed that plots the number of data points in each window that are at or below a specified value; note that regions containing many low scores will stand out as peaks in the histogram, as demonstrated by the clear association of peaks and the location of exons (Figure 1, top panel). At intermediate resolutions, a 'wiggly plot' is displayed, in which the average score for each regional window is plotted; in this case, regions containing many low scores will appear as valleys in the plot (Figure 1; middle panels). Finally, at very high resolution, the user may view the sequence data directly, along with the sequence names and a tree relating the sequences (Figure 1; lower panel).

A mobile and scalable zoom window allows for exploration of the summary views (Figure 1; upper panels). The user may drag and resize this rectangle, and when a desired region is selected a more detailed view can be obtained. This region will be expanded immediately below the parent display, with the resolution, score plot, and annotation adjusted accordingly (Figure 1; compare the start and stop coordinates of the black rectangle in the top panel to the start and stop coordinates of the entire panel immediately below). At all resolutions, annotation tracks are displayed immediately above the score/sequence display (Figure 1; all panels). An arbitrary number of tracks may be displayed, but the bottom two tracks are reserved for displaying information about transcripts with exons and introns. Colors for features can be specified individually using standard RGB notation. Other key features of the ABC include:

• Mouse-over highlighting to reveal annotations, scores, coordinates, etc

• Exporting of sequence, score, and annotation data

• Searching sequence data for particular nucleotide strings

• GoTo feature to quickly bring up a desired region

The ABC is flexible in that is has the ability to visualize diverse quantitative information and it has the capacity to display an arbitrary number of annotation types. It does not have a built-in scoring function; all data needs to be generated and formatted prior to being displayed in the ABC. While this may seem to be a drawback, it is in fact the intended function for an interface that has no preconceptions about the methodology that generated the data. Finally, the ABC is interactive, allowing the user to zoom in quickly from summary views of the comparative data to individual alignment columns. Zoom levels remain in the display, allowing the user to keep a birds-eye view of a large genomic region while focusing at much higher resolution on a small section within it.

## Conclusions

The ABC is stand-alone alignment browsing software that is relatively easy to use and customize. While it was not designed as a genome-wide browser, it is well-suited for tasks associated with comparative sequence analysis: exploration of alignments of individual genomic loci; analyzing the relationship between known biological features and quantitative comparative data; visualizing results for researchers who develop and test methods for comparative sequence analysis; isolating sequence elements in a genomic locus for downstream applications like motif-discovery or primer design; generating graphics that characterize a multiple sequence alignment or region of an alignment; and potentially more applications that we have not yet considered. In our own research, for example, we have used it to display SNPs between different mouse strains in the context of a comparative alignment (not shown). This flexibility should be beneficial to researchers whose primary interest is comparative sequence analysis, but should also be valuable to those who use comparative analyses in support of other types of projects, such as experimental characterization of constrained elements. We also note that this flexibility distinguishes the ABC from other browsers that require built-in or specific types of score data and/or the use of a particular alignment program. The software is written in Java for cross-platform support, and the source code is freely available under the General Public License (GPL).

## Availability and requirements

Project name: Application for Browsing Constraints (ABC)

Project home page: 

Operating system: Platform independent

Programming language: Java

Other requirements: Java 1.4 or later

License: GPL

## Abbrevations

ABC: Application for Browsing Constraints

GUI: Graphical User Interface

GPL: General Public License

SNP: Single Nucleotide Polymorphism

UCSC: University of California, Santa Cruz

RGB: Red-Green-Blue

## Authors' contributions

GMC and AS conceived of the project, organized the feel and design of the browser, generated the underlying data, and wrote the manuscript. SAGS wrote the Java code and provided comments on the manuscript. All authors read and approved the final manuscript.
